# Gene therapy for alopecia in type II rickets model rats using vitamin D receptor-expressing adenovirus vector

**DOI:** 10.1038/s41598-023-45594-2

**Published:** 2023-10-28

**Authors:** Satoko Kise, Ayano Iijima, Chika Nagao, Tadashi Okada, Miyu Nishikawa, Shinichi Ikushiro, Tomoko Nakanishi, Shigeto Sato, Kaori Yasuda, Toshiyuki Sakaki

**Affiliations:** 1 Department of Pharmaceutical Engineering, Faculty of Engineering, Toyama Prefectural University, 5180 Kurokawa, Imizu, Toyama 939-0398 Japan; 2 Department of Food and Nutrition, Okayama Gakuin University, 787 Aruki, Kurashiki, Okayama 710-8511 Japan; 3 Center of Biomedical Research Resources, Juntendo University School of Medicine, Juntendo University, 2-1-1 Hongo, Bunkyo, Tokyo 113-8421 Japan

**Keywords:** Genetics, Molecular biology

## Abstract

Type II rickets is a hereditary disease caused by a mutation in the vitamin D receptor (VDR) gene. The main symptoms of this disease are bone dysplasia and alopecia. Bone dysplasia can be ameliorated by high calcium intake; however, there is no suitable treatment for alopecia. In this study, we verified whether gene therapy using an adenoviral vector (AdV) had a therapeutic effect on alopecia in *Vdr*-KO rats. The VDR-expressing AdV was injected into six 7-week-old female *Vdr*-KO rats (VDR-AdV rats). On the other hand, control-AdV was injected into 7-week-old female rats (control-AdV rats); non-infected *Vdr*-KO rats (control rats) were also examined. The hair on the backs of the rats was shaved with hair clippers, and VDR-AdV or control-AdV was intradermally injected. Part of the back skin was collected from each rat after AdV administration. Hair follicles were observed using hematoxylin and eosin staining, and VDR expression was examined using immunostaining and western blotting. VDR-AdV rats showed significant VDR expression in the skin, enhanced hair growth, and low cyst formation, whereas control-AdV and non-infected rats did not show any of these effects. The effect of VDR-AdV lasted for nearly 60 days. These results indicate that gene therapy using VDR-AdV may be useful to treat alopecia associated with type II rickets, if multiple injections are possible after a sufficient period of time.

## Introduction

Vitamin D_3_ is first hydroxylated at the C25 position by CYP2R1 and CYP27A1 in the liver and then at the 1α position by CYP27B1 in the kidney to produce 1α,25-dihydroxyvitamin D_3_ (1,25D3), its active form. After 1,25D3 binds to the vitamin D receptor (VDR) in the cytoplasm, VDR translocates into the nucleus and forms a heterodimer with the retinoid X receptor (RXR). This heterodimer binds to the vitamin D response element (VDRE) on genomic DNA to regulate the expression of various genes involved in bone formation, hair follicle formation, cell differentiation, immune response, etc. When a mutation occurs in the gene encoding VDR, the aforementioned VDR-dependent vitamin D actions do not occur, causing symptoms such as bow legs/knock knees due to osteodystrophy, and alopecia due to hair follicle dysplasia; this condition is called vitamin D-dependent type II rickets (VDDRII)^1–4^. Bone dysgenesis can be ameliorated by symptomatic treatment with high calcium intake; however, no appropriate treatment for alopecia has been developed. Teichert et al.^5^ reported that treatment of *Vdr*-KO mice with an agonist to the hedgehog signaling pathway partially restored hair follicle cycling. However, its effects were only limited. Recently, we generated rat models of human type II rickets, including *Vdr*-KO and VDR(R270L) rats^6,7^. As expected, the *Vdr*-KO rats showed abnormal bone formation and alopecia (Supplemental Fig. 1). A high-Ca diet alleviated abnormal bone formation; however, it did not improve alopecia. In this study, we administered gene therapy to *Vdr*-KO rats. Gene therapy requires a normal gene (cDNA) and a carrier that delivers this gene to the patient’s cells. In this study, we used rat *Vdr* cDNA as the former and an adenoviral vector (AdV) as the latter. Viral vectors are widely used as carriers in gene therapy; they are typically non-replicating viruses in which the gene responsible for viral replication, such as the *E1A* gene of the adenovirus, is deleted. AdV can proliferate in HEK293 cells, which express the *E1A* gene. Commonly used viral vectors for gene therapy include retrovirus, lentivirus, adenovirus, and adeno-associated virus (AAV) vectors. AAV is currently the most popular viral vector in the field of gene therapy owing to its low immunogenicity^8–10^. AdV has a very high infection efficiency compared to other viral vectors and can harbor relatively large genes. However, adenoviral vectors are not integrated into the genome, thus, their effects are short-lived. In addition, multiple administrations are usually impossible due to the production of neutralizing antibodies against adenovirus proteins. Therefore, attempts have been made to extend the expression period by using immunosuppressants in combination^11^. In this study, we used the low-immunogenicity AdV developed by Nakai et al.^12^. Our final goal was to develop genome-editing therapy for alopecia using AdV with the CRISPR/Cas9 system. Once a gene is normalized by genome editing, its effects become almost permanent. A disadvantage of AdV is that its expression is transient, and the effect of gene therapy is not sustained. However, in the case of genome editing, the non-permanent expression of Cas9 using AdV is advantageous^13^. This study explored the efficacy of AdV as a preliminary step in genome-editing therapy.

**Figure 1:**
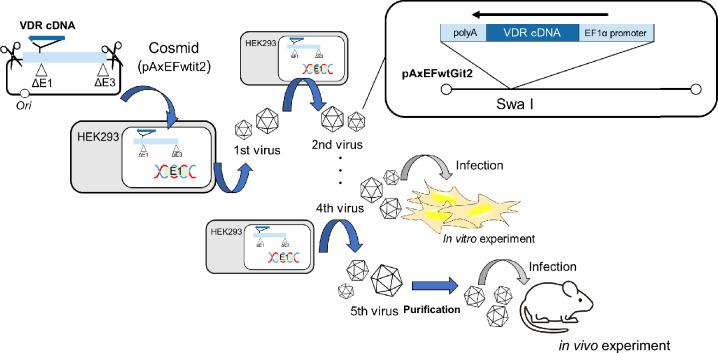
Structure of adenovirus vector for rat VDR and outline of production of the recombinant adenovirus vector in HEK293 cells. ⊿E1 and ⊿E3 mean deletions of E1 and E3 genes of adenovirus genome, respectively. pAxEFwtit2 and pAxEFwtGit2 are the names of cassete cosmids used in the construction of the recombinant adenovirus vector.

## Results

Figure 1 shows outline of production of the recombinant AdVs in HEK293 cells.

**Figure 2:**
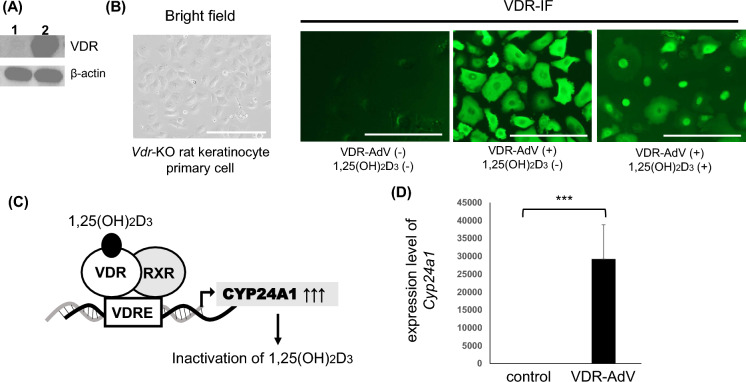
Effects of infection of VDR-AdV to keratinocyte primary cells prepared from *Vdr*-KO rats on expression of VDR protein, and induction of *Cyp24a1* mRNA by adding 1,25D3. (**A**) Western blot analysis of VDR in the VDR-AdV noninfected (lane 1) and infected (lane 2) keratinocyte primary cell of *Vdr*-KO rats. (See Supplemental Fig. 4). (**B**) Immunostaining of VDR in the VDR-AdV noninfected (VDR-AdV ( −)) and infected (VDR-AdV ( +)) cells, and nuclear translocation of VDR by adding 10 nM 1,25D3 in the cell culture (VDR-AdV( +) 1,25(OH)_2_D_3_( +)). Scale bar; 200 µm. (**C**) Outline of the mechanism of VDR-mediated induction of *Cyp24a1*. (**D**) Transcriptional induction of *Cyp24a1* by adding 10 nM 1,25D3 in VDR-AdV infected *Vdr*-KO rat primary cells. (n = 3, *p* = 0.00441).

To examine the infectivity of the obtained VDR-AdV, primary cultured cells from the skin of *Vdr*-KO rats were grown in keratinocyte growth medium and infected with VDR-AdV at a multiplicity of infection (MOI) of 10. At 24 h after infection, VDR expression was examined using western blot analysis. As shown in Fig. 2, VDR was successfully expressed in *Vdr*-KO primary keratinocytes, with an apparent molecular weight of 54 kDa. VDR was highly expressed in the VDR-AdV infected cells, whereas it was not detected in the non-infected *Vdr*-KO cells. In addition, immunostaining revealed that VDR was expressed in most VDR-AdV-infected cells. Nuclear translocation of VDR occurs upon ligand binding. Therefore, when 1,25(OH)_2_D_3_ was added to the medium of VDR-AdV-infected cells at a final concentration of 10 nM, nuclear translocation of VDR was observed in most cells (Fig. 2B). The promoter region of the *CYP24A1* gene has two VDR-binding sequences (VDRE), and the transcription of this gene is markedly enhanced by 1,25D3. At 5 h after the addition of 10 nM 1,25(OH)_2_D_3_, qPCR was performed to quantify CYP24A1 mRNA. Induction more than 30,000 times higher than that without 1,25(OH)_2_D_3_ was observed. These results strongly suggest that VDR-AdV efficiently infects *Vdr*-KO rat keratinocytes to produce high amounts of functional VDR. Therefore, when VDR-AdV is injected into the skin of *Vdr*-KO rats, VDR may function efficiently in keratinocytes.

### Effects of VDR-AdV on hair growth in *Vdr*-KO rats

**Figure 3:**
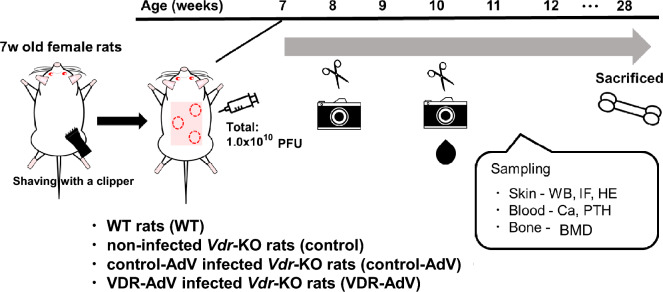
Time schedule of infection of VDR-AdV or control-AdV to *Vdr*-KO rats and evaluation of its effects in the skin (hair growth and cyst formation), blood, and bone.

Figure 3 shows a time schedule of infection of VDR-AdV or control-AdV to *Vdr*-KO rats and evaluation of its effects.

**Figure 4:**
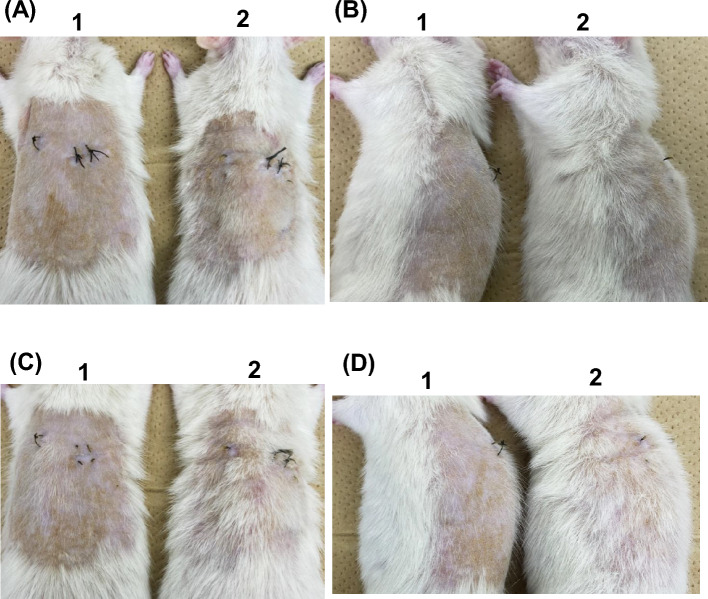
Comparison of hair growth after shaving back hair between VDR-AdV noninfected (control, lane 1) and infected (lane 2) *Vdr*-KO rats on 19 days (**A**, **B**) and 23 days (**C**, **D**) after infection. The black threads were used for suturing after cutting the skin. No. 1 and 2 correspond to control-2 and VDR-AdV-2 in Supplemental Fig. 2, respectively.

As shown in our previous report^6^, *Vdr*-KO rats exhibited alopecia, which has also been reported to occur in *Vdr*-KO mice. VDR-AdV-injected *Vdr*-KO rats showed remarkable hair growth 10 days after VDR-AdV injection, and this effect peaked around 17–30 days after injection (Fig. 4, Supplementary Fig. 2). Unexpectedly, hair growth promotion was observed over a large area and not only in the intradermal injection area, which was marked. Hence, we assumed that AdV spread out from the injection site. To determine how AdV spreads after intradermal injection, we injected GFP-AdV into *Vdr*-KO rats after skin infected with GFP-AdV turned transparent. As expected, GFP expression was observed over a large area beyond the intradermal injection site (Supplemental Fig. 3). These results strongly suggest that intradermally injected VDR-AdV also spreads in *Vdr*-KO rat skin to express VDR and promote hair growth.

### Effects of VDR-AdV on cyst formation in dermis of *Vdr*-KO rats

**Figure 5:**
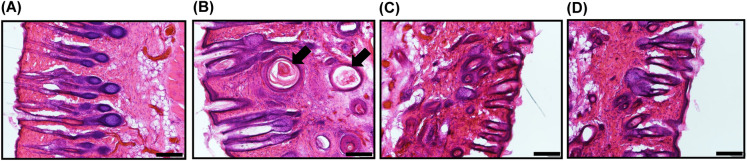
HE staining of the skin of WT (**A**), noninfected *Vdr*-KO (**B**), VDR-AdV-1–1 (**C**), and VDR-AdV-1–2 (**D**) rats in supplemental Fig. 2 on 10 days after VDR-AdV injection. The arrows in (**B**) show cyst formation. Scale bar; 200 µm.

Although one of the most prominent symptoms of type II rickets is alopecia, cyst formation was also observed in the dermis of *Vdr*-KO rats, probably due to the disfunction of hair follicles^5^. Cyst formation was evaluated by hematoxylin and eosin (HE) staining 10 days after VDR-AdV intradermal injection (59 days after birth). New cyst formation was suppressed in VDR-AdV-injected rats compared to that in both non-injected (control) and control-AdV-injected rats (Fig. 5). These results suggest that the VDR expressed in *Vdr*-KO rats by VDR-AdV suppressed cyst formation.

### Expression of VDR in skin of VDR-AdV-injected rats

**Figure 6:**
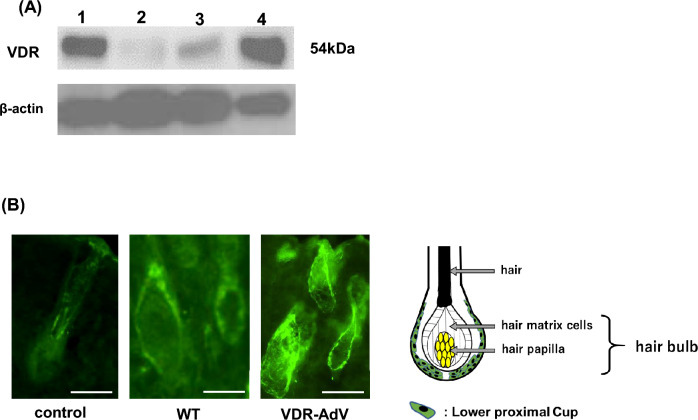
Western blot analysis (**A**) and immunostaining analysis (**B**) of VDR. (**A**) WT (lane 1), noninfected *Vdr*-KO (lane 2), VDR-AdV infected *Vdr*-KO rats (lane3; VDR-AdV-1–1, lane 4; VDR-AdV-1–2 in Supplemental Fig. 2 on 10 days after VDR-AdV injection. (See Supplemental Fig. 5). (**B**) Non-infected *Vdr*-KO (Control), WT, VDR-AdV infected *Vdr*-KO rat (VDR-AdV-1–2 in Supplemental Fig. 2 on 23 days after VDR-AdV injection. Scale bar; 200 µm.

The expression of VDR in skin was examined by western blotting and immunostaining 10 and 23 days after VDR-AdV infection, respectively. Prominent VDR expression was observed in the back skin of the VDR-AdV-injected rats (Fig. 6A, B). A faint band was also seen in the control (Fig. 6A). Although it does not seem to be derived from VDR, this band was also quantified and analyzed (Suppl. Fig. 6). The relative VDR expression level in VDR-Adv rats varies considerably depending on the individual rat and the number of days after VDR-AdV administration (2, 10, or 23 days). However, the average value was not so different from that of the WT rats (Suppl. Fig. 6). The expression levels of VDR in VDR-AdV rats were either lower or higher than that in WT rats; however, even in the former case, the amount expressed was sufficient to promote hair growth. VDR immunostaining indicated no VDR expression in the lower proximal cup (LPC) region of hair follicles in non-infected *Vdr*-KO rats (Fig. 6 B). However, remarkable VDR expression was observed in the LPC region of VDR-AdV-injected rats, as well as in WT rats^14,15^. Our results showing the localization of VDR in the LPC region suggest that VDR is stable in this region, probably because of stable complex formation with other transcription factors.

### Enhancement of *Lef1* gene expression in VDR-AdV-injected area of *Vdr*-KO rats

**Figure 7:**
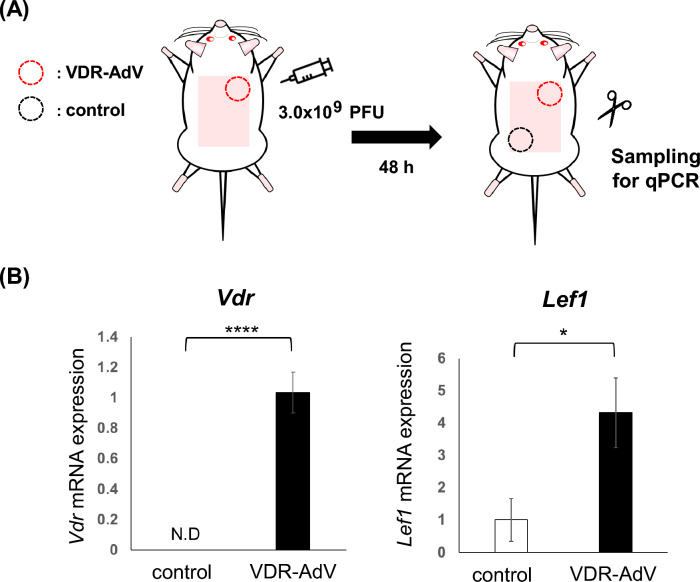
Comparison of *Vdr* and *Lef*1 gene expression between was enhanced in the skin area VDR-AdV injected compare to VDR-Ad non-injected part. (n = 3, *Vdr* mRNA: *p* = 2.04 × 10^–8^, *Lef* 1 mRNA: *p* = 0.0103).

In *Vdr*-KO rats, the expression of *Lef1* (a Wnt signaling-related gene) at the VDR-AdV-infected site was approximately fourfold that at the uninfected site (Fig. 7). As mentioned above, AdV spreads over a considerable range when intradermally injected on the back. Therefore, a site far from the infected site was selected as the control. In addition, VDR-AdV-dependent *Vdr* mRNA at each site was comparatively quantified to confirm that VDR-AdV had no influence on the expression away from the injection site. Since the *Vdr* mRNA expressed by VDR-AdV has a modified sequence with regard to codon usage, the primer set used (Supplemental Table 1) did not detect natural rat *Vdr* mRNA.

### Effects of VDR-AdV on plasma Ca levels and bone mineral density (BMD) in *Vdr*-KO rats

Plasma Ca levels and BMD in VDR-AdV-injected and non-injected (control) *Vdr*-KO rats were compared. No significant differences were observed in these parameters between the two groups (Supplemental Fig. 7).

## Discussion

For type II rickets based on *Vdr*-KO, bone formation can be normalized by excess Ca intake; however, there is no therapeutic drug or methodology for alopecia. Hence, we explored the possibility of using gene- and genome-editing therapies for treating alopecia caused by VDR deficiency. Several studies have demonstrated that complex formation by VDR and other factors in keratinocytes is essential for the maintenance of the hair cycle^16,17^. In addition, the interaction between Hairless and the VDR-RXR complex may be essential for the regulation of hair follicle cycling, including the hedgehog signaling pathway^5,18^. Furthermore, it has been reported that VDR may interact with RXR, Lef1, Hairless, and β-catenin to activate Wnt signals and promote the proliferation of keratinocyte stem cells present in the bulge region of hair follicles^5,17–19^. A novel model of hair follicle development was recently reported^20^; however, the role of VDR is unknown.

In this study, *Vdr*-KO rats (40% deletion of the C-terminus of VDR and deletion of most of the ligand-binding region) were used as therapeutic targets. As stated in our previous report, *Vdr*-KO rats exhibited alopecia and cysts on the skin. By 25 weeks of age, most of their body hair had fallen out, and they nearly resembled nude mice. Mesenchymal tissue induces the formation of hair follicles only once, when the hair is first formed; thereafter, the hair cycle stops being repeated. Thus, VDR deficiency has no effect on mesenchymal tissue-mediated hair and hair follicle formation; however, VDR is essential for the second stage of the hair cycle, suggesting that it is an important factor in this cycle.

Our experiments employed WT rats (as positive controls), non-infected *Vdr*-KO rats (control, *n* = 4), *Vdr-*KO rats infected with control-AdV (control-AdV, *n* = 2), and *Vdr*-KO rats infected with VDR-AdV (VDR-AdV, *n* = 6). The hair on the back skin of 12 animals was shaved to the same degree with clippers, and Vdr-AdV was injected into three parts of the back (Fig. 2). The VDR-AdV-infected rats showed remarkable hair growth, reduced hair loss, low cyst formation, and significant expression of VDR. To the best of our knowledge, there have been no reports on intradermal injections of recombinant AdV for gene therapy. Our results demonstrate the utility of gene therapy with AdV for alopecia caused by VDDRII. Judging from the area of hair on the head, several tens of times more VDR-AdV than that used in each *Vdr*-KO rat (1.0 × 10^10^ plaque-forming unit (PFU) may be required for intradermal injection into the hairy epidermis of a patient. However, this vector does not require large-scale equipment and can be manufactured at the laboratory level. The effect of VDR-AdV lasted for nearly 60 days without an increase in the aspartate aminotransferase (AST) value, which is an index of damage to several organs, suggesting that no severe inflammation was caused by VDR-AdV injection (data not shown). These results may be due to the use of the AdV with low immune response developed by Nakai et al.^12^. Since the plasma Ca concentration and BMD in VDR-AdV-infected rats were similar to those in control rats, the effects of VDR-AdV were limited to the skin.

As described previously, AdVs expressing VDR or each of its mutants appear to be useful for elucidating the detailed mechanism of VDR function in sustaining the hair cycle^21^. Recently, we revealed that VDR(R270L/H301Q) has almost no binding affinity for either 1,25D3 or 25D3. In addition, we generated *Vdr*(R270L/H301Q) rats using genome editing^21^. Although these rats showed abnormal bone formation, no alopecia was observed, suggesting that unliganded VDR plays an important role in hair cycle maintenance. In a *Vdr*-KO mouse study, *Lef1* expression was reduced in *Vdr*-KO mouse hair follicle cells and in skin cells with an alopecia phenotype^5^. *Lef1*-KO mice exhibited decreased hair density, hair thinning, and weakness compared to WT mice^22^. The VDR-AdV injection site showed 4 times higher gene expression of *Lef1* compared to the non-infected site in *Vdr*-KO rats (Fig. 7). Thus, the remarkable effects of VDR-AdV injection on hair growth may involve an increase in *Lef1* expression to activate the Wnt signaling pathway. Therefore, it can be assumed that complex formation between unliganded VDR and other factors such as RXR, Lef1, Hairless, and β-catenin might be essential for the activation of the Wnt signaling pathway in hair cycle maintenance.

If multiple injections are possible after a sufficient period of time, VDR-AdV may be useful for actual treatment. However, our final goal is to achieve permanent treatment by genome-editing therapy. Although it is possible to directly inject a patient with VDR-AdV, it may also be possible to extract keratinocytes from the patient, efficiently introduce VDR with AdV, increase the number of cells, and then transplant them into the patient.

## Materials and methods

### Materials

25(OH)D_3_ and 1α,25(OH)_2_D_3_ were purchased from Wako Pure Chemical Industries, Ltd (Osaka, Japan). PCR primer DNAs and pMA-based plasmid containing DNA fragment encoding wild type of rat VDR were purchased from Eurofins Genomics (Tokyo, Japan), as described previously^21^. Other chemicals were commercially available and of the highest quality.

### Preparation of primary culture cells

Back skins prepared from 7 w old *Vdr*-KO female rats were used to obtain primary keratinocyte cells. After removing a white adipose tissue, the back skins were washed with PBS, and incubated in 14 ml CnT-07 epithelial proliferation medium (CELLnTEC ADVANCED CELL SYSTEMS AG, Switzerland) with 5 mg/ml collagenase at 4 °C for 18 h. The resultant back skin sample was washed with PBS to remove extra collagenase, and incubated with 5 ml of Tryp Express (Thermo Fisher #12604013) at 37 °C for 15 min, and then 25 mL of a CnT-07 medium was added and pipetted tremendously to disassociate cell–cell adhesion. The resultant cell solution was centrifugated at 2,000 rpm for 5 min. The resultant pellet containing keratinocyte cells were suspended with 4 ml of CnT-07 medium with penicillin–streptomycin and amphotericin B with the final concentration of 100 unit/ml and 0.5 µg/ml respectively, and the harvested cells were cultured in 6 cm collagen coated dishes. At 2 days from culturing, culture medium was changed from CnT-07 medium to Keratinocyte Growth Medium 2 (PromoCell, Germany).

### Animals and diets

Jcl:Wistar rats were obtained from CLEA Japan Inc. (Tokyo, Japan). Embryonic microinjection for genome editing was performed by KAC Co., Ltd. (Kyoto, Japan).

All of rats were kept at room temperature (22–26 °C), and in 50–55% humidity with a 12 h light/dark cycle. They were allowed food and water ad libitum and fed F-2 (Oriental Yeast Co., Tokyo, Japan) containing 0.75% Ca and 2000 international unit (IU) vitamin D /kg diet^6^. Homozygotes of *Vdr-*KO were maintained by mating of homozygotes. Genotype was determined by electrophoresis of PCR products of the target site for *Vdr*-KO^6^. In this study, female rats were used based on the report describing that female *Vdr*-KO mice lose hair faster than males^23^, although we have not confirmed whether a similar phenomenon occurs in *Vdr*-KO rats.

All experimental protocols using animals were performed in accordance with the Guidelines for Animal Experiments at Toyama Prefectural University and were approved by the Animal Research and Ethics Committee of Toyama Prefectural University.

### Structure of cosmid cassette and AdV

We constructed rat VDR expressing Ad (VDR-Ad) and GFP expressing Ad (GFP-Ad) using a cosmid cassette^21^. HEK293 cells (ATCC CRL-1573, a human embryonic kidney cell line containing the Ad 5 E1 region; American Type Culture Collection [ATCC], Manassas, VA) were maintained in Dulbecco’s modified Eagle’s medium (DMEM) supplemented with 10% fetal bovine serum (FBS). AdVs were constructed using the cosmid cassette pAxcwit2 containing the full-length AdV genome^24,25^. In addition, EF1α promoter, which is originally derived from pEF321-T^26,27^ was used for expression of rat VDR. Note that we used a 2.1-kb SwaI–EcoRI fragment of this plasmid for all AdV constructions including the AdV construction cassette pAxEFwtit2 (Fig. 1), which was deposited in the RIKEN Bioresource Center DNA Bank (Tsukuba, Japan); therefore, the promoter is different from commercially available EF1α promoters, which are shorter.

### Construction of AdV expressing wild type of rat VDR, and its production and purification

The DNA fragment encoding wild type rat VDR, which was modified from viewpoint of codon usage without change of amino acid sequence, was chemically synthesized, as previously described^21^. In addition, Kozak sequence and BsrG1 site at 5’ terminus, and NsiI site at 3’ terminus was added^21^. The plasmid containing *Vdr* cDNA fragment was cleaved by BsrG1 and NsiI, and after blunting, the resultant fragment was inserted into Swa I site of the cassete cosmid pAxEFwtGit2 (Fig. 1). To produce AdV using the resultant cosmids, HEK293 cells were transfected with the BstBI-linearized AdV genome. On the next day cells were transferred to a 96-well plate, and the virus clones were obtained within 2 weeks (first viral stock of 150–200 μL). Cells in the 24-well plates were infected with one half of the first stock to obtain the second stock, as shown previously^28^. HEK293 cells in a 6 cm dish, a 9 cm dish, and two of 9 cm dishes were infected with the second virus, third virus, and fourth virus to obtain the next stage virus, respectively. Fourth virus was used for in vitro experiment, and fifth virus were used for animal experiment after purification by the adenovirus purification kit (ViraBindTM Adenovirus Purification Kit, Cell Biolabs, Inc.) according to its protocols (Fig. 1).

### Intradermal infection of AdV to *Vdr*-KO rats

The 7wk-old Vdr-KO female rats were used for animal experiments. Totally 1.0 × 10^10^ PFU of VDR-Ad were injected intradermally to the back skin of *Vdr*-KO rats (0.33 mL of 1.0 × 10^10^ PFU/mL was injected at 3 sites), and gave skin swelling a mark with marker to make easy sampling (Fig. 3). After 2 or 10 or 23 days from infection, a part of skin was taken from the area injected VDR-Ad of each rat, and conducted experiment such as WB, immunostaining, HE staining, and also observed the appearance of hair growth promotion.

### Intradermal injection of GFP-AdV to Vdr-KO rats and transparency of the skin to visualize the range of AdV infection area

5.0 × 10^8^ PFU of GFP-AdV was injected intradermally to a single site with a circle of 1 cm in diameter of a 7w-old female Vdr-KO rat (Supplemental Fig. 2). At 48 h after injection, the skin was trimmed over a wider area (approximately 2.5 cm × 3.5 cm) than the injection site, and then fixed with 4% PFA (Wako, Japan) at 4 °C for 24 h. The fixed skin was processed with CUBIC-Trial-Kit (FUJIFILM Wako Pure Chemical Coporation, Osaka, Japan,) aiming to transparency of GFP-AdV injected skin. Transparency was performed along with CUBIC-Trial-Kit instructions. In briefly, fixed skin was incubated in 50% ScalCUIBIC-1 solution for 24 h, and the solution was changed to 100% ScalCUIBIC-1 solution. After incubation at 37 °C for 6 days, the skin was washed with PBS, and incubated in 50% ScalCUIBIC-2 solution for 24 h. After changing the medium to 100% ScalCUIBIC-2 solution, the skin was incubated at room temperature for 24 h.

### Western blot analysis

The back skin was cut and homogenized with Minylis personal homogenizer (bertin technologies, Montigny-le-Bretonneux, France). The resultant tissue lysate containing 5 µg or 30 µg protein was applied to each lane of the gel, and then subjected to SDS–PAGE on 4 to 20% linear gradient polyacryl amide/SDS gels. After electrophoresis, gels were electrotransferred onto PVDF membranes. The membranes were incubated in TBS-T containing 5% skim milk, and then incubated with anti-VDR antibody (D2K6, rabbit mAb) (Cell Signaling Technology, Danvers, MA, USA)^21^. The membranes were washed three times with Tris-buffered saline containing 0.05% Tween 20 (TBS-T) and incubated with horseradish peroxidase-conjugated goat anti-rabbit IgG (Cell Signaling Technology, Danvers, MA, USA). The membranes were washed with Tris-buffered saline containing 0.05% Tween 20 (TBS-T), and then followed by enhanced chemiluminescence immunodetection method (Amersham Pharmacia Biotech, Buckinghamshire, England).

### Immunofluorescence staining

Each rat skin sample was fixed with 4% PFA (FUJIFILM Wako Pure Chemical Corporation, Tokyo, Japan) for 15 h at 4 °C. The fixed samples were mounted with O.T.C compound (Sakura Finetek Japan, Tokyo, Japan) in a container and feezed in liquid nitrogen. The frozen sections were prepared with cryostat microtome (Leica, Tokyo, Japan) thickness 20-25 µm each sample, and sticked it to glass slaids. Anti-VDR antibody (D2K6W) Rabbit mAb (Cell Signaling Technology, Danvers, MA, USA) (first antibody), and Alexa Flour 488 goat anti-rabbit IgG (Invitrogen, Carlsbad, USA) (second antibody) were used for immunofluorescence^21^. After staining nuclei with DAPI, samples were observed using phase contrast microscopy (Olympus, Tokyo Japan).

### HE staining

Each rat skin sample was fixed with 4% PFA (FUJIFILM Wako Pure Chemical Corporation, Osaka, Japan) for 15 h at 4 °C. The fixed samples were mounted with O.T.C compound (Sakura Finetek, Japan) in container and feezed in liquid nitrogen. Frozen sections were obtained by cryostat microtome (Leica, Tokyo, Japan) with thickness of 20-25 µm, and stuck to glass slides. The 4% PFA solution was dripped to the sample glass, which was incubated at room temperature for 10 min, and washed with water for 10 min. Hematoxylin was dripped on the sample glass, which was incubated at room temperature for 15 min, and washed with water for 10 min. Eosin-alcohol were dripped on the sample glass, which was incubated at room temperature for 2 min, and washed with water for 2 min, 70% EtOH for 2 min, 80% EtOH for 2 min, 90% EtOH for 2 min, 100% EtOH for 2 min, and then washed with xylene twice. The resultant HE stained samples were observed using a phase contrast microscopy (Olympus, Tokyo Japan) (Fig. 5).

### RNA isolation and qPCR analysis

*Vdr*-KO primary keratinocyte cells on a 6 cm collagen coated dish were infected with rat-Vdr- AdV at moi of 10. At 24 h after infection, 10 nM of 1,25D3 were added and after 5 h, cells were lysed in ISOGEN II (Nippon Gene, Tokyo, Japan), and then RNAs were collected along with ISOGEN II protocol. The cDNAs were synthesized using ReverTra Ace qPCR RT Master Mix with gDNA Remover (Toyobo, Osaka, Japan). PCR was performed on Real-Time PCR System (Thermal cycler Dice Real Time System III, Takara, Shiga, Japan) with SYBR green master mix, and the primers shown in Supplemental Table 1 were used for q-PCR.

### Measurement of plasma Ca concentrations

The plasma Ca concentration was measured using the Calcium E-Test Wako (Wako Pure Chemical, Osaka, Japan)^6^.

### Measurement of bone mineral density

Bone mineral density (BMD) was determined between the proximal and distal epiphysis of the left femora. After muscle removal, the left femora of the rats (n = 3 animals for each group) were scanned using an X-ray CT system (Latheta LCT-200; Hitachi Aloka Medical, Tokyo, Japan)^6^. Parameters used for the CT scans were as follows: tube voltage, 50 kVp; tube current, 500 μA; integration time, 3.6 ms; axial field of view, 48 mm, with an isotropic voxel size of 48 μm. The mineral content of the femur was calculated using LaTheta software (Hitachi Aloka Medical). A threshold density of 160 mg/cm^3^ was selected to distinguish mineralized from unmineralized tissue. The density range was calibrated daily with a manufacturer-supplied phantom^6^.

### Statistical analysis

The statistical significance of differences in Figs. 2, 7, and Suppl. Fig. 6 CYP24A1 mRNA level induced by 1,25D3 was analyzed by the Student’s t-test. The criterion for significance was *p* < 0.05.

### Ethical approval

This study was reported in accordance with ARRIVE guidelines (https://arriveguidelines.org).

### Supplementary Information

Supplementary Information.

## Data Availability

The datasets generated or analyzed during the current study are available from the corresponding author (T.S.) on reasonable request. A genomic sequence of *Vdr* gene of *Vdr* KO rats containing the mutated position is available in DDBJ data base accession number LC764592 (http://getentry.ddbj.nig.ac.jp/top-j.html).

## References

[1] Malloy PJ, Feldman D (2011). The role of vitamin D receptor mutations in the development of alopecia. Mol. Cell Endocrinol..

[2] Feldman D, Malloy PJ (2014). Mutations in the vitamin D receptor and hereditary vitamin D-resistant rickets. Bonekey. Rep..

[3] Arita K, Nanda A, Wessagowit V, Akiyama M, Alsaleh QA, McGrath JA (2008). A novel mutation in the VDR gene in hereditary vitamin D-resistant rickets. Br. J. Dermatol..

[4] Tamura M, Ishizawa M, Isojima T, Özen S, Oka A, Makishima M, Kitanaka S (2017). Functional analyses of a novel missense and other mutations of the vitamin D receptor in association with alopecia. Sci. Rep..

[5] Teichert A, Elalieh H, Bikle D (2010). Disruption of the hedgehog signaling pathway contributes to the hair follicle cycling deficiency in Vdr knockout mice. J. Cell Physiol..

[6] Nishikawa M, Yasuda K, Takamatsu M, Abe K, Okamoto K, Horibe K, Mano H, Nakagawa K, Tsugawa N, Hirota Y, Horie T, Hinoi E, Okano T, Ikushiro S, Sakaki T (2020). Generation of novel genetically modified rats to reveal the molecular mechanisms of vitamin D actions. Sci. Rep..

[7] Nishikawa M, Murose N, Mano H, Yasuda K, Isogai Y, Kittaka A, Takano M, Ikushiro S, Sakaki T (2022). Robust osteogenic efficacy of 2α-heteroarylalkyl vitamin D analogue AH-1 in VDR (R270L) hereditary vitamin D-dependent rickets model rats. Sci. Rep..

[8] Nishiguchi KM, Fujita K, Miya F, Katayama S, Nakazawa T (2020). Single AAV-mediated mutation replacement genome editing in limited number of photoreceptors restores vision in mice. Nat. Commun..

[9] Richards DY, Winn SR, Dudley S, Nygaard S, Mighell TL, Grompe M, Harding CO (2019). AAV-mediated CRISPR/Cas9 gene editing in murine phenylketonuria. Mol. Ther. Methods Clin. Dev..

[10] Hakim CH, Kumar SRP, Pérez-López DO, Wasala NB, Zhang D, Yue Y, Teixeira J, Pan X, Zhang K, Million ED, Nelson CE, Metzger S, Han J, Louderman JA, Schmidt F, Feng F, Grimm D, Smith BF, Yao G, Yang NN, Gersbach CA, Chen SJ, Herzog RW, Duan D (2021). Cas9-specific immune responses compromise local and systemic AAV CRISPR therapy in multiple dystrophic canine models. Nat. Commun..

[11] Yamamoto K, Yuasa K, Miyagoe Y, Hosaka Y, Tsukita K, Yamamoto H, Nabeshima YI, Takeda S (2000). Immune response to adenovirus-delivered antigens upregulates utrophin and results in mitigation of muscle pathology in mdx mice. Hum. Gene Ther..

[12] Nakai M, Komiya K, Murata M, Kimura T, Kanaoka M, Kanegae Y, Saito I (2007). Expression of pIX gene induced by transgene promoter: Possible cause of host immune response in first-generation adenoviral vectors. Hum. Gene Ther..

[13] Boucher P, Cui X, Curiel DT (2020). Adenoviral vectors for in vivo delivery of CRISPR-Cas gene editors. J. Control Release.

[14] Sequeira I, Nicolas JF (2012). Redefining the structure of the hair follicle by 3D clonal analysis. Development.

[15] Yang H, Adam RC, Ge Y, Hua ZL, Fuchs E (2017). Epithelial-mesenchymal micro-niches govern stem cell lineage choices. Cell.

[16] Chen CH, Sakai Y, Demay MB (2001). Targeting expression of the human vitamin D receptor to the keratinocytes of vitamin D receptor null mice prevents alopecia. Endocrinology.

[17] Bouillon R, Carmeliet G, Verlinden L, Etten EV, Verstuyf A, Luderer HF, Lieben L, Mathieu C, Demay M (2008). Vitamin D and human health: Lessons from vitamin D receptor null mice. Endocr. Rev..

[18] Hsieh JC, Sisk JM, Jurutka PW, Haussler CA, Slater SA, Haussler MR, Thompson CC (2003). Physical and functional interaction between the vitamin D receptor and hairless corepressor, two proteins required for hair cycling. J. Biol. Chem..

[19] Luderer HF, Gori F, Demay MB (2011). Lymphoid enhancer-binding factor-1 (LEF1) interacts with the DNA-binding domain of the vitamin D receptor. J. Biol. Chem..

[20] Morita R, Sanzen N, Sasaki H, Hayashi T, Umeda M, Yoshimura M, Yamamoto T, Shibata T, Abe T, Kiyonari H, Furuta Y, Nikaido I, Fujiwara H (2021). T racing the origin of hair follicle stem cells. Nature.

[21] Kise S, Iijima A, Nagao C, Okada T, Mano H, Nishikawa M, Ikushiro S, Kanemoto Y, Kato S, Nakanishi T, Sato S, Yasuda K, Sakaki T (2023). Functional analysis of vitamin D receptor (VDR) using adenovirus vector. J. Steroid Biochem. Mol. Biol..

[22] Zhou P, Byrne C, Jacobs J, Fuchs E (1995). Lymphoid enhancer factor 1 directs hair follicle patterning and epithelial cell fate. Genes Dev..

[23] Li YC, Pirro AE, Amling M, Delling G, Baron R, Bronson R, Demay MB (1997). Targeted ablation of the vitamin D receptor: An animal model of vitamin D-dependent rickets type II with alopecia. Proc. Natl. Acad. Sci. U S A.

[24] Miyake S, Makimura M, Kanegae Y, Harada S, Sato Y, Takamori K, Tokuda C, Saito I (1996). Efficient generation of recombinant adenoviruses using adenovirus DNA-terminal protein complex and a cosmid bearing the full-length virus genome. Proc. Natl. Acad. Sci. U S A.

[25] Fukuda H, Terashima M, Koshikawa M, Kanegae Y, Saito I (2006). Possible mechanism of adenovirus generation from a cloned viral genome tagged with nucleotides at its ends. Microbiol. Immunol..

[26] Kim DW, Uetsuki T, Kaziro Y, Yamaguchi N, Sugano S (1990). Use of the human elongation factor 1 alpha promoter as a versatile and efficient expression system. Gene.

[27] Kim DM, Harada T, Saito I, Miyamura T (1993). An efficient expression vector for stable expression in human liver cells. Gene.

[28] Nakanishi T, Maekawa A, Suzuki M, Sato K, Abata H, Mori M, Saito I (2021). Construction of adenovirus vectors simultaneously expressing four multiplex, double-nicking guide RNAs of CRISPR/Cas9 and in vivo genome editing. Sci. Rep..

