# Supplementary material for: Gene therapy for alopecia in type II rickets model rats using vitamin D receptor-expressing adenovirus vector
Source: Sci Rep. 2023 Oct 28;13:18528. doi: 10.1038/s41598-023-45594-2 (PMC10613246; doi:10.1038/s41598-023-45594-2)
Supplement: Supplementary file 1 — Supplementary Information. [file 41598_2023_45594_MOESM1_ESM.pdf]

Scientific Reports

Gene therapy for alopecia in type II rickets model rats using vitamin D receptor-expressing adenovirus vector

Satoko Kise[1], Ayano Iijima[1], Chika Nagao[1], Tadashi Okada[2], Miyu Nishikawa[1], Shinichi Ikushiro[1], Tomoko Nakanishi[3], Shigeto Sato[3], Kaori Yasuda[1], Toshiyuki Sakaki[1]*

1.Department of Pharmaceutical Engineering, Faculty of Engineering, Toyama Prefectural University, 5180 Kurokawa, Imizu, Toyama 939-0398, Japan.
2. Department of Food and Nutrition, Okayama Gakuin University, 787 Aruki, Kurashiki, Okayama 710-8511, Japan.
3. Center of Biomedical Research Resources, Juntendo University School of Medicine, Juntendo University, 2-1-1 Hongo, Bunkyo, Tokyo 113-8421

Running title: Gene therapy for alopecia using adeno virus vector

＊Address correspondence to : 5180 Kurokawa, Imizu, Toyama 939-0398, Japan.

Fax: +81-766-56-2498; E-mail: tsakaki@pu-toyama.ac.jp

Key words: gene therapy, vitamin D receptor, adeno virus vector, rickets, alopecia

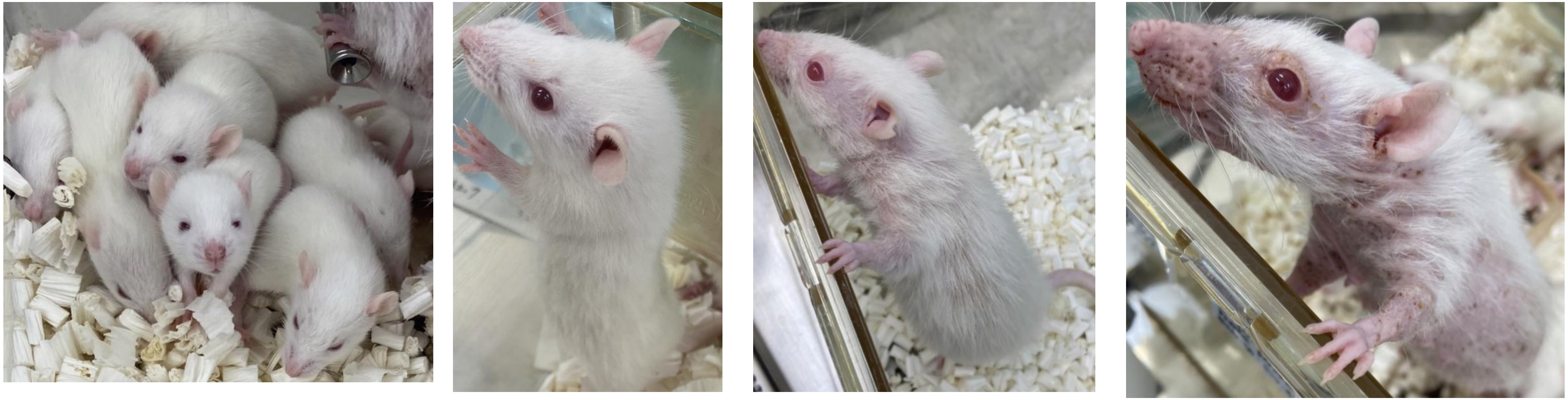

(A) 2w

(B) 6w

(C) 12w

(D) 24w

Supplemental Fig. 1

Alopecia phenotype of VDR-KO rats with their aging.

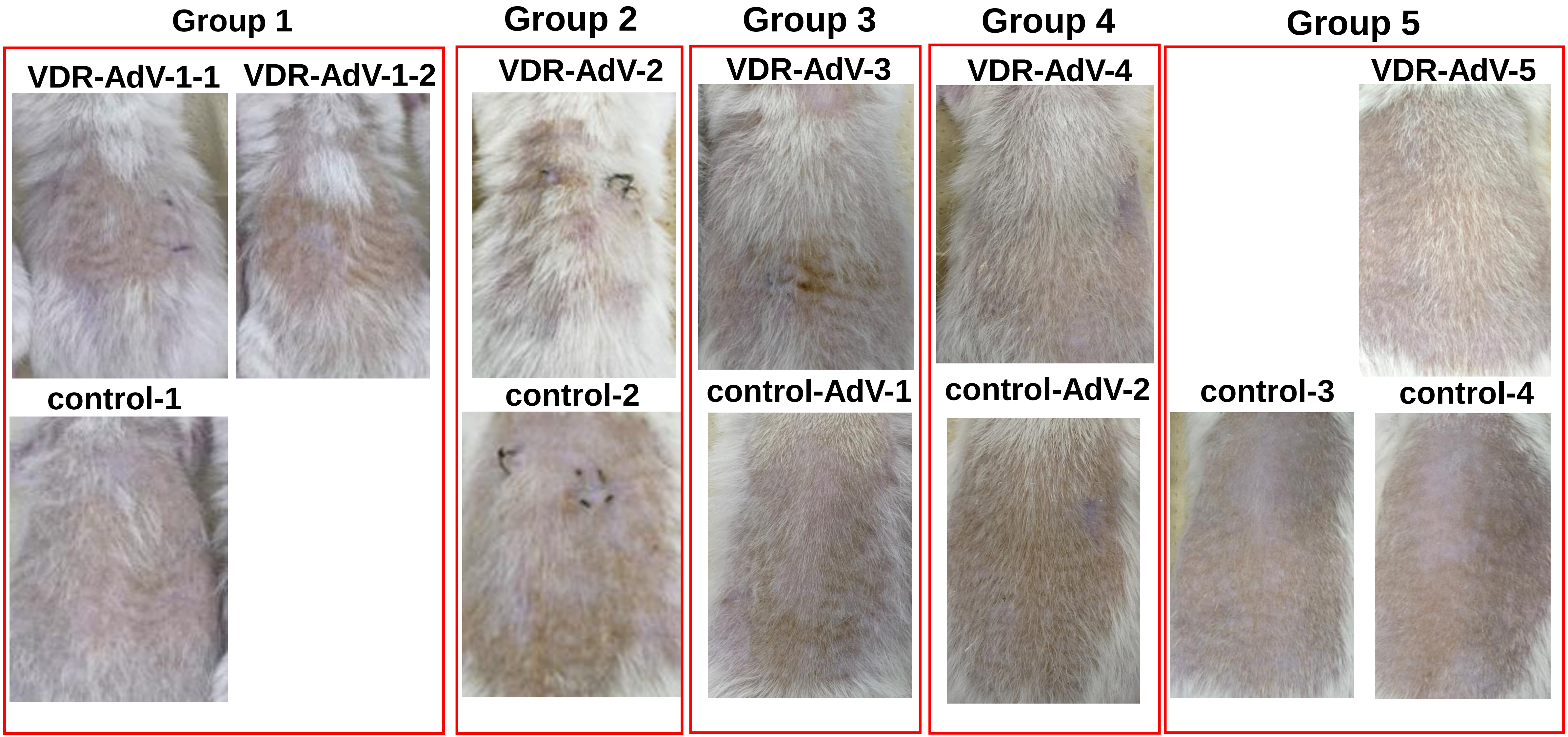

Supplemental Fig.2

Comparison of hair growth among VDR-Ad infected, non-infected (control), and control-AdV infected Vdr-KO rats. Animal experiments were performed 5 times designated as Group 1 to 5. In the first experiment, 2 rats were infected with VDR-AdV, and 1 rat was non-infected. Conversely, in the fifth experiment, one animal was infected with VDR-AdV and two were non-infected. The black threads observed in Group 1 and 2 were used for suturing after cutting the skin

Group 1：After 30 days from back-hair shaved
Group 2：After 23 days from back-hair shaved
Group 3：After 30 days from back-hair shaved
Group 4：After 26 days from back-hair shaved
Group 5：After 17 days from back-hair shaved

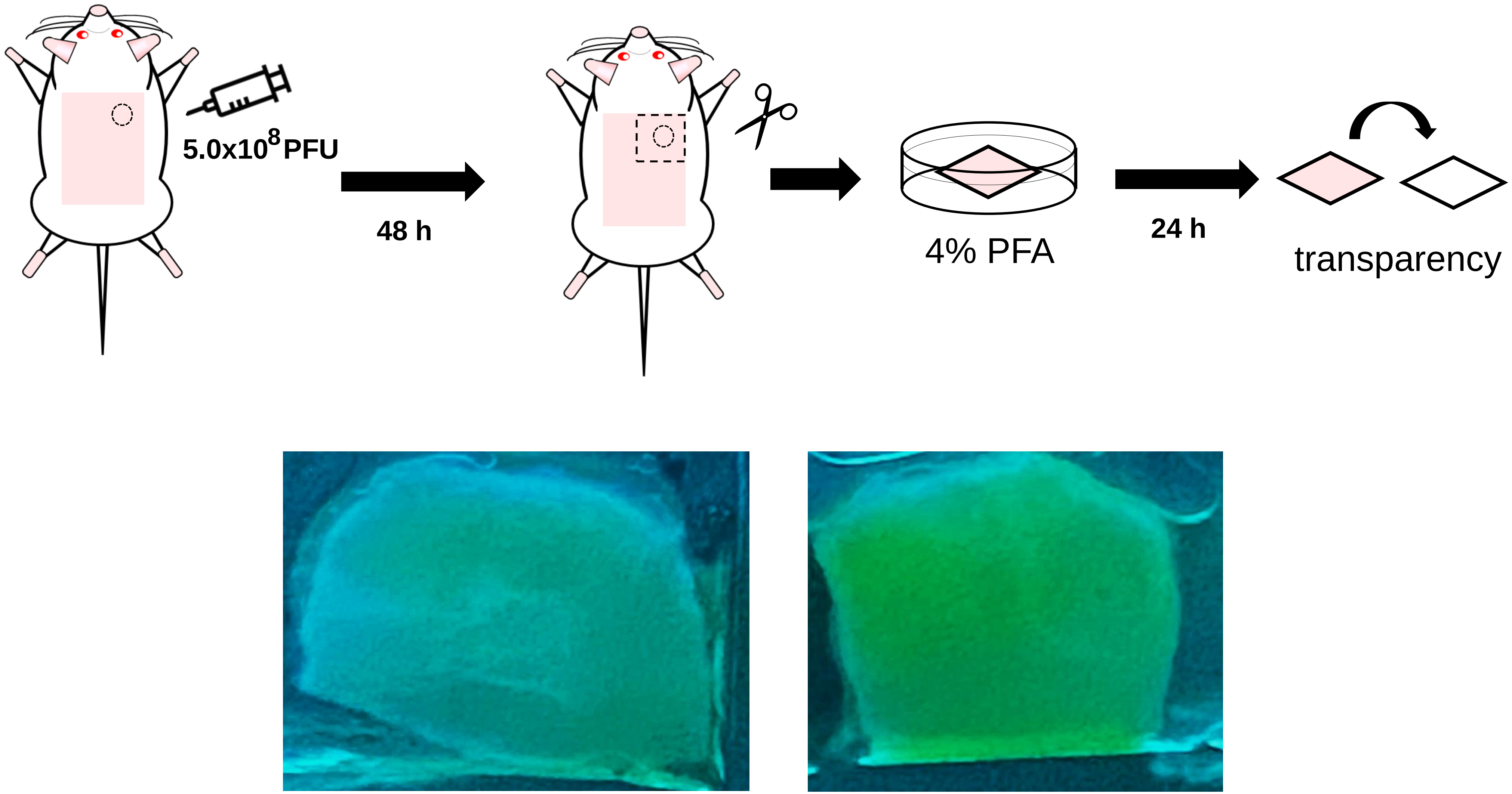

Supplemental Fig. 3
GFP expression in the skin infected with GFP-AdV after transparency treatment.
The circle and rectangle mean the GFP-AdV infected area, and cut out part, respectively.

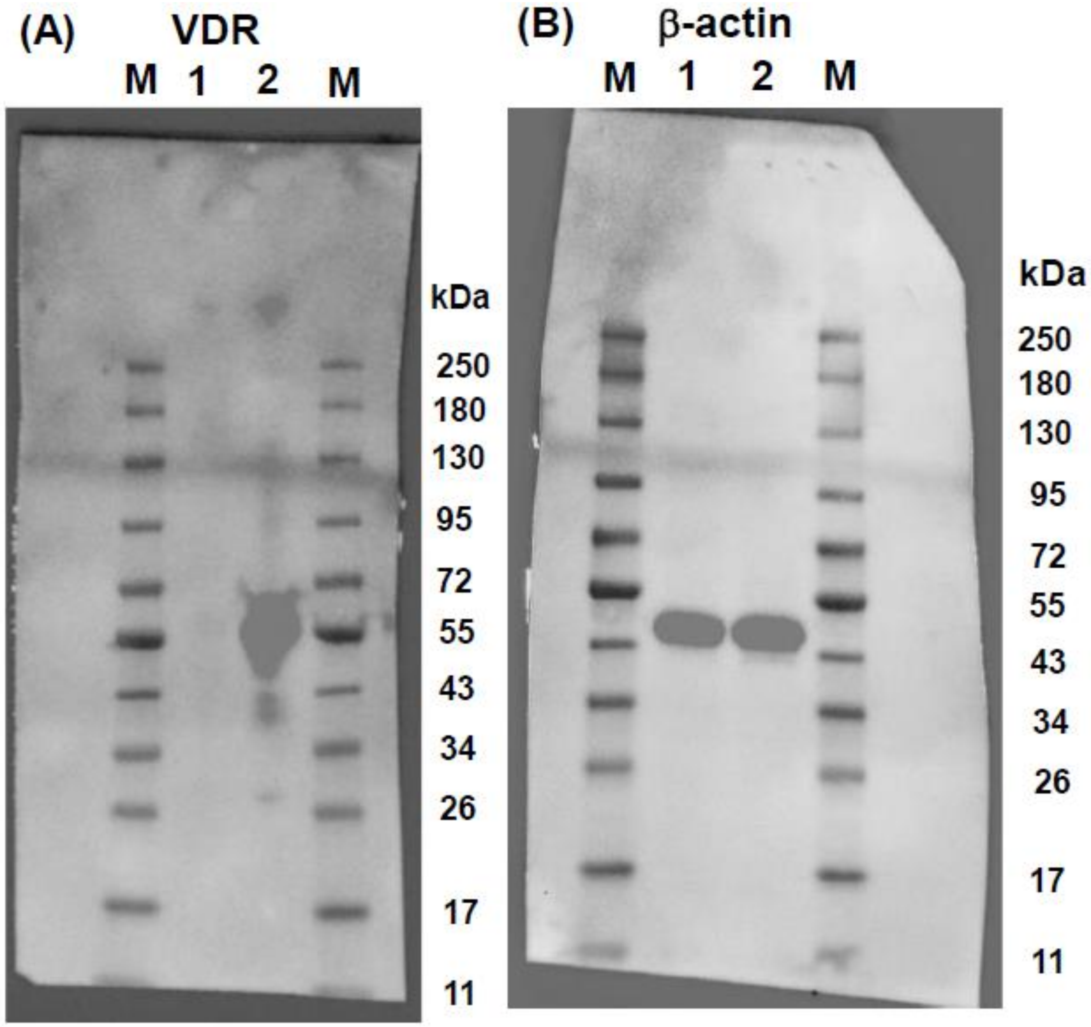

Supplemental Fig. 4
Western blot analysis of VDR (A) and β-actin (B) shown in Fig.3A with molecular size marker proteins (Blue Pre-stained protein Standard, Broad Range P7718S, New England BioLabs. Inc. MA, USA) VDR-AdV noninfected (lane 1) and infected (lane 2) keratinocyte primary culture cells prepared from *Vdr*-KO rats.

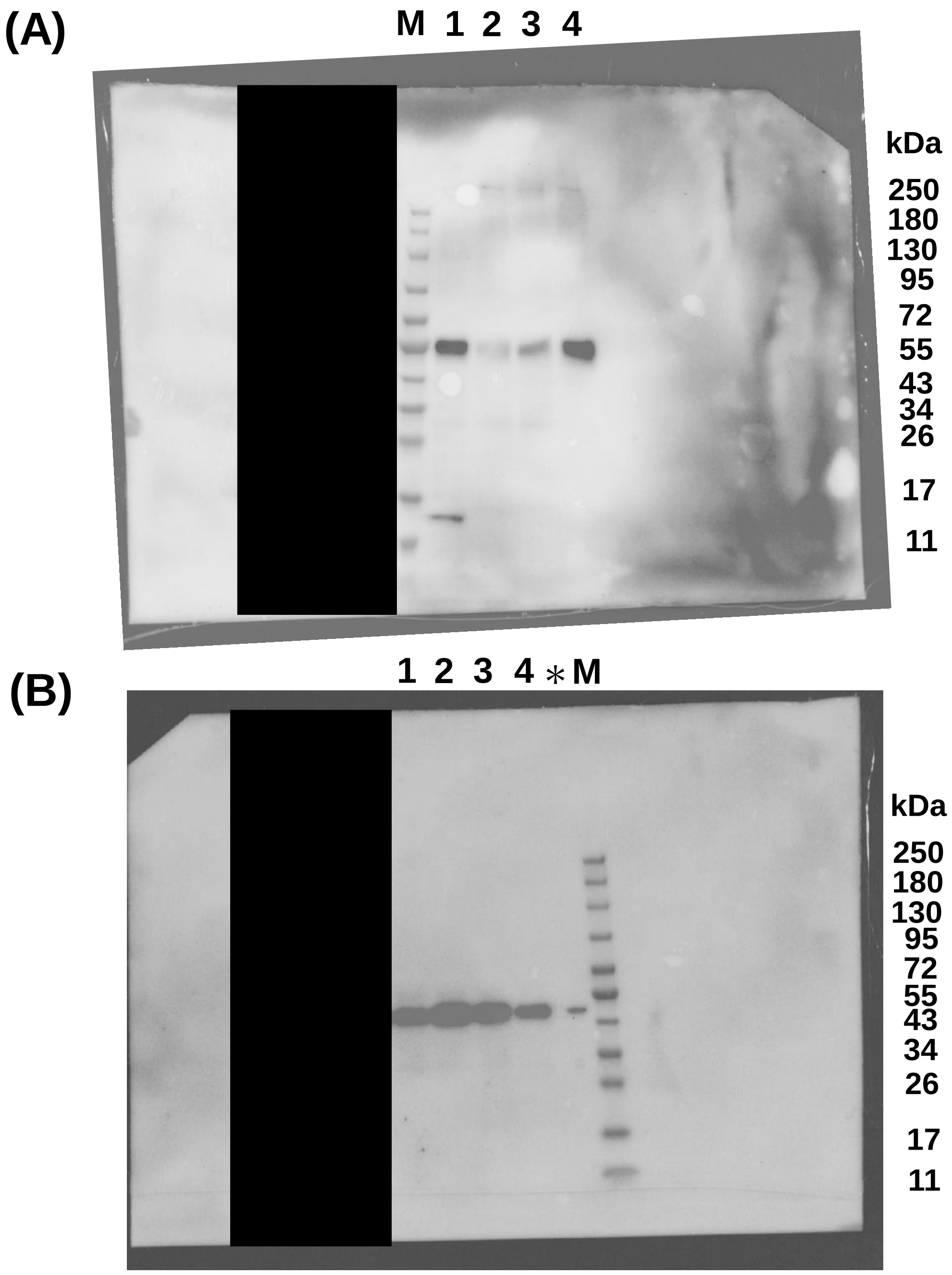

Supplemental Fig. 5
Western blot analysis of VDR (A) and β-actin (B) shown in Fig.6 with molecular size marker proteins.
lane 1, WT; lane 2, noninfected *Vdr*-KO; lane 3, VDR-AdV infected *Vdr*-KO rat (VDR-AdV-1-1 in supplemental Fig.2); lane 4, VDR-AdV-1-2 in supplemental Fig.2 on 10 days after VDR-AdV injection.
* A small amount of the sample No. 4 was contaminated in this lane.

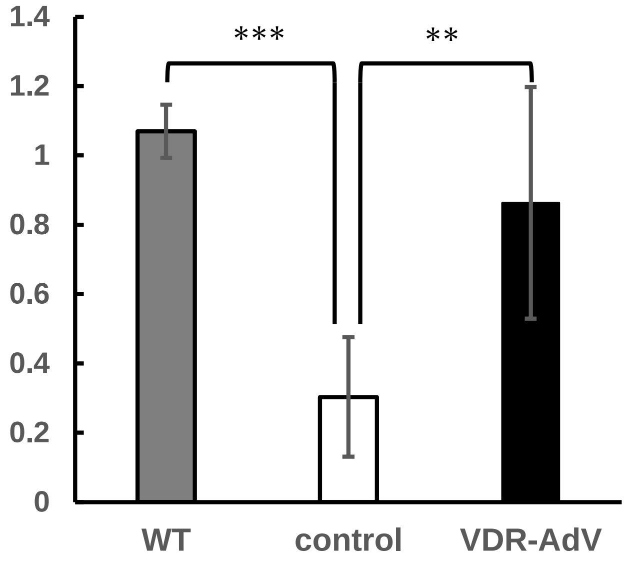

Supplemental Fig. 6

The relative expression levels of VDR/β-actin were compared.

WT rats (mean ± SD =1.070 ± 0.077, n=3),

non-infected *Vdr*-KO rats (control) (mean ± SD =0.303 ± 0.172, n=4),

VDR-AdV infected *Vdr*-KO rats (mean ± SD =0.863 ± 0.334, n=7)

The significant differences between the groups are as follows.

WT vs control (p=0.00087, ***: P<0.001)

control vs VDR-AdV (p=0.00996, **: P<0.01)

WT vs VDR-Adv (p=0.18536)

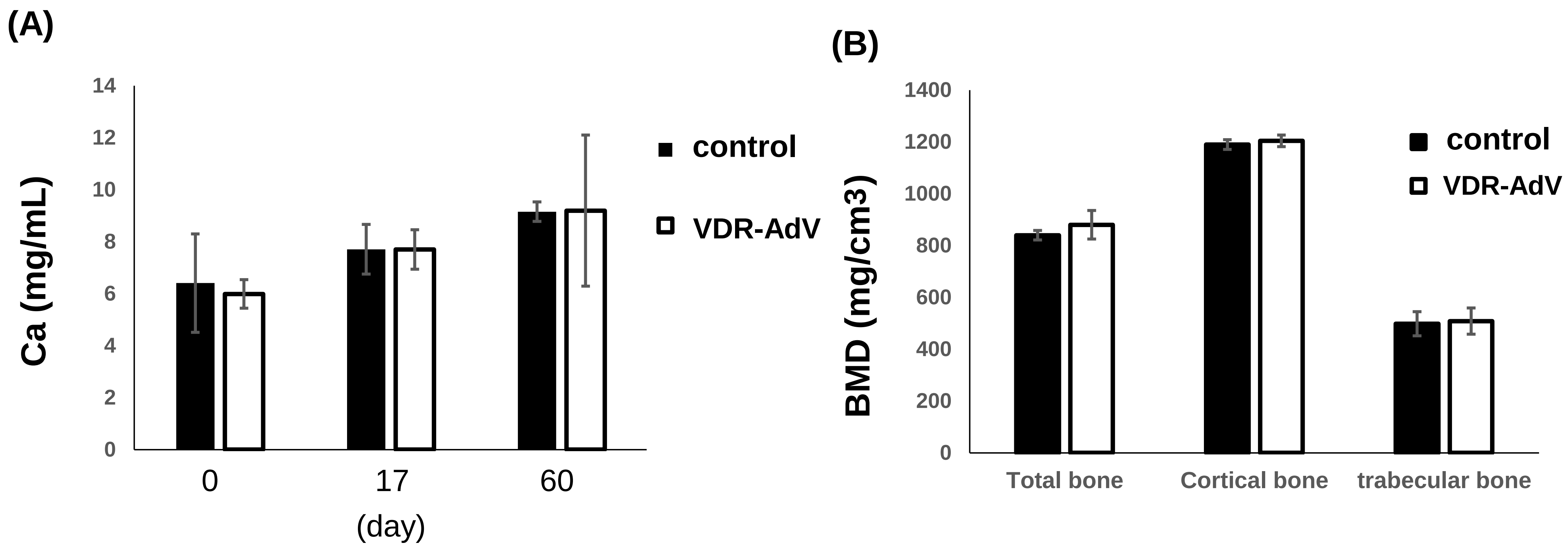

Supplemental Fig.7

Comparison of plasma Ca (A) and bone mineral density (B) between VDR-Ad infected and non-infected Vdr-KO rats (n=3).

Supplemental Table 1　PCR primer sets used for qPCR

rat *Cyp24A1*	Forward　5'-AGCCCGGGGCAGATTTCCTCTG-3'<br>Reverse　5'-CATATTCCTCAGGTCTTCCGC-3'
rat β-actin	Forward　5'- AGGCCCAGAGCAAGAGAGGCAT -3'<br>Reverse　5'- CATATCGTCCCAGTTGGTGACA -3
rat *Lef-1*	Forward　5'- CACACAACTGGCATCCCTCATC -3'<br>Reverse　5'- GCTCCTGTTCCTTTCTCTGTTCGT -3
rat *Vdr* derived from VDR-AdV	Forward　5'- AGGAGCACGTCCTGCTAATG -3'<br>Reverse　5'- GTGTCGGCACCGGATATAGG -3
